# In Vitro Protective Effects of a Standardized Extract of *Opuntia ficus-indica* (L.) Mill. Cladodes and *Olea europaea* L. Leaves Against Indomethacin-Induced Intestinal Epithelial Cell Injury

**DOI:** 10.3390/antiox13121507

**Published:** 2024-12-10

**Authors:** Federica Lina Salamone, Maria Sofia Molonia, Claudia Muscarà, Antonella Saija, Francesco Cimino, Antonio Speciale

**Affiliations:** Department of Chemical, Biological, Pharmaceutical and Environmental Sciences, University of Messina, Viale F. Stagno D’Alcontres 31, 98166 Messina, Italy; federica.salamone@studenti.unime.it (F.L.S.); mmolonia@unime.it (M.S.M.); cmuscara@unime.it (C.M.); asaija@unime.it (A.S.); specialea@unime.it (A.S.)

**Keywords:** indomethacin, NF-κB, Caco-2, oxidative stress, apoptosis, anti-inflammatory activity, tight junctions, *Opuntia ficus-indica*, *Olea europaea*

## Abstract

Nonsteroidal anti-inflammatory drugs (NSAIDs) can induce serious adverse effects in gastrointestinal (GI) mucosa, increasing intestinal permeability and leading to mitochondrial dysfunction, oxidative stress, apoptosis and inflammation. As proton pump inhibitors are effective in protecting against NSAID-induced gastropathy but not NSAID-induced enteropathy, current research is focused on natural products as protective substances for therapy and prevention of intestinal injury. Herein, through the use of an in vitro model based on intestinal epithelial cell (Caco-2) damage caused by indomethacin (INDO), we examined the protective activity of a commercially available standardized extract (OFI+OE) from *Opuntia ficus-indica* (L.) Mill. cladodes and *Olea europaea* L. leaves. Pre-treatment with OFI+OE prevented INDO-induced intestinal epithelial barrier damage, as demonstrated by TEER measurement, fluorescein permeability, and tight junction protein expression. The extract showed positive effects against INDO-induced oxidative stress and correlated activation of apoptosis, decreasing pro-apoptotic markers BAX and Caspase-3 and increasing anti-apoptotic factor Bcl-2. Moreover, the extract inhibited the NF-κB pathway and pro-inflammatory cascade. In conclusion, these data support the use of OFI+OE extract as a natural strategy for therapy and prevention of intestinal mucosal damage, demonstrating its beneficial effects against INDO-induced intestinal damage, through modulation of oxidative, apoptotic, and inflammatory pathways.

## 1. Introduction

Nonsteroidal anti-inflammatory drugs (NSAIDs) are broadly employed for their antipyretic, analgesic, and anti-inflammatory effects exerted through inhibition of cyclooxygenase (COX) activity [1]. Unfortunately, these drugs are recurrently associated with severe adverse events mainly affecting gastrointestinal (GI) mucosa, such as erosions and ulcers. Indeed, the chance of GI damage with high doses or chronic use of NSAIDs is an important concern [2].

Indomethacin (INDO) is an NSAID with broad applications [3] and, due to its high ulcerogenic activity, is the most used drug in ulcer models [4], and is also used to assess the protective activity of bioactive molecules [5]. The pathogenesis of enteropathy induced by INDO is still unclear, but it has been reported to uncouple mitochondrial oxidative phosphorylation in enterocytes, inhibiting oxygen consumption, dissipating mitochondrial membrane potential, and decreasing intracellular ATP [6,7]. INDO has been shown to alter paracellular intestinal permeability, mainly affecting the integrity of epithelial tight junctions (TJs), composed of transmembrane proteins (Occludins and Claudins), peripheral membrane proteins (Zonula occludens), and subjacent adherens junctions (E-cadherin), between adjacent cells [2,8]. Increased permeability of the intestinal epithelial barrier, in turn, exposes cells to the cytotoxic luminal contents, such as bile, proteolytic enzymes, pancreatic secretions and intestinal bacteria, that may reach the mucosa, inducing inflammation through neutrophils infiltration and oxidative stress by generation of reactive oxygen species (ROS) [9]. INDO-induced oxidative stress is probably a triggering mechanism inducing cell death via dysfunction of endoplasmic reticulum (ER) and inhibition of mitochondrial complex I activity [10,11].

Although proton pump inhibitors (PPIs) have largely resolved the problem of NSAID-induced gastropathy [12], they are unable to protect from NSAID-induced enteropathy, which may instead be exacerbated [13]. Strategies of therapy against NSAID-induced damage are often represented by mucoadhesive and mucoprotective substances and by treatment of related inflammatory and cytotoxic consequences [14,15,16,17]. It can also be hypothesized that molecules with antioxidant potential are able to alleviate intracellular stress and prevent cell death [18]. Therefore, research is currently directed at protective substances for therapy and prevention of NSAID-induced GI injury, especially natural products, preferred for their biocompatibility and safety properties. In fact, interest in plant-derived compounds is constantly increasing due to their wide range of activities, such as anti-inflammatory, antioxidant, anticancer, immunomodulatory, and antimicrobial properties [19,20,21]. In particular, polyphenols have shown beneficial effects against intestinal diseases, so that they may be taken into account as new agents for the prevention and treatment of these pathologies [22].

The prickly pear cactus *Opuntia ficus-indica* (L.) Mill. (Cactaceae family) is a tropical and subtropical species usually growing in dry zones, such as in the Mediterranean and in Central America [23]. This shrub-like plant has various flat branches named cladodes [24,25] which are rich in mucilage [26,27], a carbohydrate hydrocolloid with a very branched structure. This intricate biopolymer is constituted of a variety of neutral sugars, such as D-galactose, D-xylose, L-arabinose, D-galacturonic acid, and L-rhamnose, mainly linked to uronic acids [28,29,30]. Pharmaceutical industries have shown increasing interest in *Opuntia* mucilage [31], particularly recently, due to its various physicochemical, thermal, rheological, and functional properties. In addition, polysaccharides from *O. ficus-indica* cladode extracts may be very likely involved in gastrointestinal mucosa regeneration [32], and can be applied as mucoprotective compounds thanks to their ability to form a molecular network and retain water [23]. In fact, as suggested by the beneficial effects observed in rats with ethanol-induced ulcers, *Opuntia* polysaccharides have been shown to possess mucoprotective activity primarily due to their bioadhesive properties on epithelial mucosa and ability to enhance mucus production [33,34,35]. Vázquez-Ramírez et al. [36] hypothesized that the beneficial action of *O. ficus-indica* cladode mucilage against experimental ethanol-induced gastric damage might be linked to the stabilization of plasma membranes in injured gastric mucosa, as a result of interactions between monosaccharides present in mucilage and phospholipids in the membrane. Interestingly, Rizza et al. [37] demonstrated using an in vitro model of Caco-2 cell monolayers that *Opuntia* cladode polysaccharides possess a better mucoadhesivity than hyaluronic acid, validating the hypothesis that they can attach to the mucosal cell surface and create a physical barrier. Furthermore, incubation of Caco-2 cell cultures with bacterial lipopolysaccharide (LPS) caused an upregulation of ICAM-1 that was significantly reduced by *Opuntia* cladode polysaccharides.

Furthermore, *Opuntia* cladode extracts contain also polyphenols, piscidic acid being one of the most abundant [38,39]. Piscidic acid, a phenolic acid rich in hydroxyl groups, acts as an iron chelator with strong antioxidant properties [40], and its diffusion is rare in nature, mainly found in crassulacean acid metabolism and succulent plants. Its presence in *Opuntia* cladodes extract seems to significantly contribute to its capability to in vitro protect keratinocytes against UV light-induced damage [41].

Instead, *Olea europaea* L. (Oleaceae family) is an evergreen tree growing in the Mediterranean basin. Olive leaves represent a natural source of polyphenolic molecules, and their extracts have been widely used in traditional medicine for preventing and treating various disorders, regarding immune and cardiovascular systems, and as an antimicrobial agent [42,43]. The most widespread polyphenols in olive leaf extracts are luteolin and apigenin as well as oleuropein, an iridoide monoterpene which is probably responsible for the biological activities of these products [44,45]. Scientific evidence supports that olive polyphenols have the ability to protect the mucosa due to their antioxidant and radical scavenger activities, and can modulate inflammatory responses related to mucosal diseases [37,46]. Furthermore, Dekanski and colleagues [44] also demonstrated its antioxidant effect on gastric mucosal damage in rats.

The aim of this study was to investigate the in vitro protective effects of a commercially available standardized extract from *O. ficus-indica* (L.) Mill. cladodes and *O. europaea* L. leaves (OFI+OE). Herein, we employ a model of intestinal epithelial Caco-2 cell injury induced by INDO to evaluate the protective effects of the extract on the intestinal mucosa. We focused on its impact on the functionality of the intestinal epithelial barrier, intracellular redox status, and modulation of inflammatory and apoptotic pathways.

## 2. Materials and Methods

### 2.1. Reagents

In this study, we tested a commercially available standardized extract (product batch number 05202210-06) called Mucosave^TM^ FG (OFI+OE), kindly provided by Bionap S.R.L (Belpasso, Catania, Italy). It is composed of *Opuntia ficus-indica* (L.) Mill. cladodes juice extract and *Olea europaea* L. leaves aqueous extract, with 45% (*w*/*w*) of maltodextrin as support. Total polyphenols content determined by HPLC is 3.9% (*w*/*w*) (Figure S1, Table S1), while total polysaccharides content, determined using the phenol–sulphuric method, is 27% (*w*/*w*). Details about the used OFI+OE extract and HPLC-DAD polyphenolic profile are available in the Supplementary Materials.

Indomethacin (INDO) was purchased from Alfa Aesar (Thermo Fisher, Kandel, Germany). Dimethyl sulfoxide (DMSO) was purchased from AppliChem (Darmstadt, Germany). All other reagents, unless otherwise specified, were acquired from Sigma-Aldrich (Milan, Italy).

### 2.2. Cell Cultures and Treatments

Caco-2 human epithelial cells, obtained from American Tissue Culture Collection (ATCC), were grown in Dulbecco’s modified eagle’s medium (DMEM) supplemented with 10% FBS, 4 mM L-glutamine, 1% non-essential amino acids, 100 U/mL penicillin and 100 μg/mL streptomycin. Cells were maintained at 37 °C in a humidified atmosphere with 95% air and 5% CO_2_ and medium was changed every 2 days. To better reproduce in vivo intestinal conditions, Caco-2 monolayers were prepared by seeding cells at 4 × 10^4^ per cm^2^ on the upper side of transwell inserts (0.4 μm pore size; Greiner Bio-One, Cassina de’ Pecchi, Milano, Italy) of 12-well plates (Greiner Bio-One, Italy) and cultured for 18 days post-confluence to obtain fully differentiated cells [47,48]. All the experiments were carried out on fully differentiated cells. Monolayer integrity and formation of TJs were evaluated by measurement of Trans-Epithelial Electrical Resistance (TEER) by using a Millicell-ERS Voltohmeter (Millipore, Burlington, MA, USA). Monolayers used in the study had TEER values ≥ 600 Ω·cm^2^.

Fully differentiated Caco-2, prepared as described above, was pre-treated or not for 24 h with OFI+OE and added to the apical side of the transwell inserts. OFI+OE was always freshly dissolved in DMEM and used immediately. At the end of the incubation time, cells were washed twice with Dulbecco’s phosphate-buffered saline (DPBS) with calcium and magnesium in both compartments, exposed for 24 h to INDO 1 mM (final concentration of DMSO 0.1% *v*/*v*), and added to the apical compartment of the permeable filter support. Cells treated with vehicle alone (DMSO 0.1%) in DMEM were used as control (CTR). Both OFI+OE and INDO were added only to the apical side of the transwell inserts to mimic oral administration. For all the experiments, two OFI+OE concentrations were selected (350 and 700 μg/mL), since concentrations lower than 350 μg/mL were not effective (did not significantly improve TEER values compared to the cells exposed to INDO), while concentrations higher than 700 µg/mL showed slight cytotoxic effects (Figure S2).

### 2.3. TEER Determination

Barrier integrity was evaluated by TEER (Trans-Epithelial Electrical Resistance) assessment, using a Millicell-ERS Voltohmeter (Millipore, MA, USA). TEER was measured at the end of all treatments and values were expressed as Ω·cm^2^.

### 2.4. Fluorescein Permeability

In order to evaluate the intestinal epithelial barrier function, the paracellular permeability of the Caco-2 cells monolayer was determined after OFI+OE and INDO treatments, using sodium fluorescein as a marker. Cells were washed in both compartments with DPBS and then 0.5 mL of a solution (100 μM) of sodium fluorescein, solubilized in DMEM without phenol red, was added to the apical side of the insert, while 1 mL of DMEM without phenol red was added to the basolateral compartment. Caco-2 cells were incubated at 37 °C for 1 h, and finally, the basolateral compartment solutions were collected. Fluorescence was measured through a fluorimeter (FLUOstar Omega Plate Reader, BMG LABtech, Ortenberg, Germany) at 490 nm excitation and 514 nm emission.

The apparent permeability coefficient (P_app_) was calculated from the following equation:

$$P_{app}\left(\frac{cm}{s}\right)=\frac{V}{AC_{0}}*\frac{dC}{dt}$$

where dC/dt is the change in concentration at the basolateral side over time (μM/s), V is the volume of solution in the basolateral compartment (1 mL), A is the surface area of the membrane (1.12 cm^2^), and C_0_ is the initial concentration at the apical side (100 μM). The results were expressed as a percentage of apparent permeability (P_app_) versus control [49].

### 2.5. Cells Lysate Extraction

After the treatments, cells were rinsed with DPBS without calcium and magnesium and harvested with trypsin-EDTA. Whole cell lysate was prepared in non-denaturing lysis buffer (10 mM Tris HCl, pH 7.4, 150 mM NaCl, 1% Triton X-100, and 5 mM EDTANa_2_) containing protease inhibitors (1 μg/mL leupeptin, 1 mM benzamidine, and 2 μg/mL aprotinin) and 1 mM dithiothreitol. Nuclear and cytoplasmatic extracts were prepared as previously described [50]. At the end, protein fractions were stored at −80 °C until use. Protein concentration in lysates was assessed by the Bradford reagent (Bradford, 1976), using bovine serum albumin (BSA) as standard.

### 2.6. Western Blot Analysis

For immunoblotting analysis, 30 μg of protein lysates per sample were denatured in 4x SDS-PAGE sample buffer (260 mM Tris-HCl, pH 8.0, 40% (*v*/*v*) glycerol, 9.2% (*w*/*v*) SDS, 0.04% bromophenol blue, and 2-mercaptoethanol as reducing agent), and then subjected to SDS-PAGE on 10 or 12% acrylamide/bisacrylamide gels. In order to determine the NF-κB (nuclear factor kappa light-chain-enhancer of activated B cells) p65 nuclear level, nuclear lysates were used. Caspase-3 levels were evaluated in whole-cell lysates, whereas Claudin-1, Occludin, phosphorylated 5′ adenosine monophosphate-activated protein kinase (pAMPK), BAX, and Bcl-2 were evaluated in cytoplasmatic lysates. Separated proteins were transferred to the PVDF membrane (Hybond-P PVDF, Amersham Bioscience, Buckinghamshire, UK). Residual binding sites on the membrane were blocked by incubation in TBST (10 mM Tris, 100 mM NaCl, 0.1% Tween 20) with 5% (*w*/*v*) non-fat milk powder for 1 h at room temperature. Membranes were probed overnight at 4 °C with specific primary antibodies: rabbit anti-NF-kB p65 monoclonal antibody (Cell Signaling Technology, Danvers, MA, USA) (1:1000); rabbit anti-Claudin-1 monoclonal antibody (Cell Signaling Technology) (1:2000); mouse anti-Occludin monoclonal antibody (Santa Cruz Biotechnology, Dallas, TX, USA) (1:500); rabbit anti-Phospho-AMPKα (Thr172) monoclonal antibody (Cell Signaling Technology) (1:2000); rabbit anti-BAX monoclonal antibody (Cell Signaling Technology) (1:1000); rabbit anti-Bcl2 policlonal antibody (1:1000) (Sigma-Aldrich); mouse anti-Caspase-3 monoclonal antibody (1:500) (Santa Cruz Biotechnology); rabbit anti-β-Actin monoclonal antibody (Cell Signaling Technology) (1:6000); and rabbit anti-Lamin-B monoclonal antibody (Cell Signaling Technology) (1:1500). Then, membranes were incubated for 2 h at 4 °C with peroxidase-conjugated secondary antibody: anti-rabbit Ig (Cell Signaling Technology, Danvers, MA, USA) (1:6000) or anti-mouse Ig (Cell Signaling Technology) (1:6000). The luminescence was visualized with an ECL plus detection system (Amersham Biosciences) or with Clarity Max ECL (Bio-Rad, Hercules, CA, USA). Blots were detected using high-performance chemiluminescence film (Amersham Hyperfilm™ ECL; GE Healthcare Life Sciences, Buckinghamshire, UK) or using a ChemiDoc Imaging System (Bio-Rad, Hercules, CA, USA). Protein loading homogeneity was evidenced by Ponceau staining and housekeeping proteins β-Actin or Lamin-b. Quantitative analysis was performed by densitometry using the softwares ImageJ (v1.54g) and Image Lab (Bio-Rad, Hercules, CA, USA).

### 2.7. Real-Time PCR

Total cellular RNA was isolated using the E.Z.N.A. Total RNA Kit (OMEGA Bio-Tek VWR, Invitrogen), quantified with Quant-iT™RNA assay kit by QUBIT fluorometer (Invitrogen, Milan, Italy), and reverse transcripted with the M-MLV reverse transcriptase. Real-time PCR (polymerase chain reaction) was performed on a 7300 Real-Time PCR System (Applied Biosystems, Monza, Italy) coupled with SYBR green chemistry (SYBR green JumpStart^TM^ Taq Ready Mix, Sigma). The genes identified were *IL-6* (FW 5′-GATGGATGCTACCAAACTGGAT-3′, RV 5′-CCAGGTAGCTATGGTACTCCAGA-3′) [51], *COX-2* (FW 5′-ATGCTGACTATGGCTACAAAAGC-3′, RV 5′-TCGGGCAATCATCAGGCAC-3′) (PrimerBank ID: 223941909c3) [52], and *TNF-α* (FW 5′-CCAGGC AGTCAGATCATCTTCTC-3′, RV 5′-AGCTGGTTATCTCTCAGCTCCAC-3′) [48]. *18S* rRNA (FW 5′-GTAACCCGTTGAACCCCATT-30, RV 50-CCATCCAATCGGTAGTAGCG-3′) [53] was used as a reference gene. The fold increase in mRNA expression, compared with the control cells treated with the vehicle only, and corrected with the *18S* rRNA housekeeping gene, was determined using the 2^−ΔΔCt^ method [54].

### 2.8. Intracellular Total Antioxidant Activity (TAA)

Following appropriate treatments, cells were collected and homogenized in Triton X-100 0.05%. TAA in cell lysates was evaluated by decolouration of the radical cation of 2,2-azinobis-(3-ethybenzothiazoline-6-sulfonic acid) (ABTS), in terms of absorbance quenching at 734 nm [55]. This assay determines the capacity of antioxidants to quench the ABTS^+^ radical [56]. Each sample was added to 2 mL of ABTS radical solution and then incubated for 6 min in the dark at room temperature. Finally, the absorbance was recorded using a UV-vis spectrophotometer (Shimadzu, Japan), with Trolox used as standard. All determinations were carried out in triplicate and repeated three times. The results were expressed as nmoles of Trolox equivalents/mg of proteins [57] using Trolox, a water-soluble vitamin E analog, for the calibration curve.

### 2.9. ROS Measurement by Dichlorodihydro-Fluorescein Diacetate Assay

Generation of ROS was measured by the oxidation-sensitive fluorescent probe, dichloro-dihydro-fluorescein diacetate (DCFH-DA), as a modified method previously described [57]. After the opportune treatments, cells were washed three times with DPBS without calcium and magnesium at both the compartment of trans-well insert and treated with DCFH-DA 50 μM into the apical side at 37 °C for 30 min in the dark. Then, DCFH-DA was removed, and cells were washed with DBPS (pH 7.4) to remove the excess probe. Fluorescence was measured at 485 and 530 nm (excitation and emission, respectively) using a fluorimeter (FLUOstar Omega Plate Reader, BMG LABtech, Ortenberg, Germany). The fluorescence intensity is directly proportional to the ROS amount. ROS levels were expressed as DCFH-DA relative fluorescence intensity and reported as fold versus control. Each analysis was carried out in triplicate.

### 2.10. Statistical Analysis

All the experiments were carried out on three experimental units (*n* = 3), each performed in triplicate. Data were statistically analysed by a one-way or a two-way ANOVA test, followed by Tukey’s HSD, using the statistical software ezANOVA v.0.98 (https://people.cas.sc.edu/rorden/ezanova/index.html, accessed on 6 March 2023). Differences in groups and treatments were considered significant for *p* < 0.05.

## 3. Results

### 3.1. Protective Effects of OFI+OE on Indomethacin-Induced Intestinal Epithelial Barrier Function Alteration

**Figure 1:**
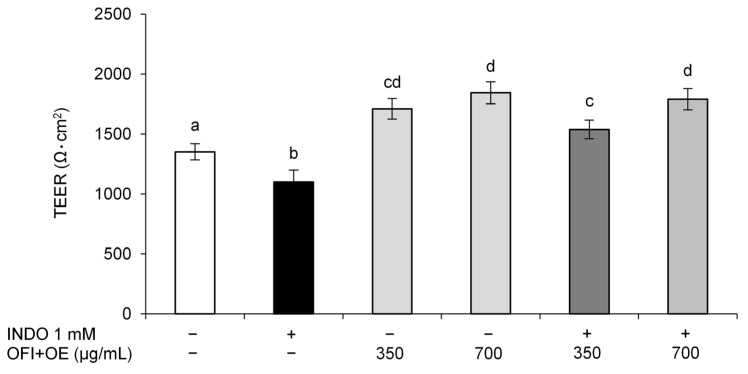
TEER evaluation. Fully differentiated Caco-2 cells were pre-treated with OFI+OE (350 and 700 μg/mL) for 24 h and then exposed to INDO 1 mM for 24 h. At the end of the treatments, TEER was determined. Cells treated with the vehicle alone (DMSO 0.1%) were used as controls. Results are reported as Ω·cm^2^ and expressed as mean ± SD of three independent experiments. Means with the same letter are not significantly different from each other (*p* > 0.05).

Previous studies showed that INDO induces cell injury in the intestinal epithelial barrier by altering cell permeability and disassembling TJ proteins [9]. The functionality of differentiated Caco-2 cells was investigated by TEER measurement after OFI+OE and INDO treatments. The results demonstrated that OFI+OE (350 and 700 μg/mL) was able to protect cells from INDO-induced intestinal epithelial barrier function alteration in a dose-dependent manner, as shown by TEER increase compared to cells treated with INDO (Figure 1). Furthermore, OFI+OE improved the intestinal epithelial barrier function in Caco-2 cells not incubated with INDO in comparison with control cells.

**Figure 2:**
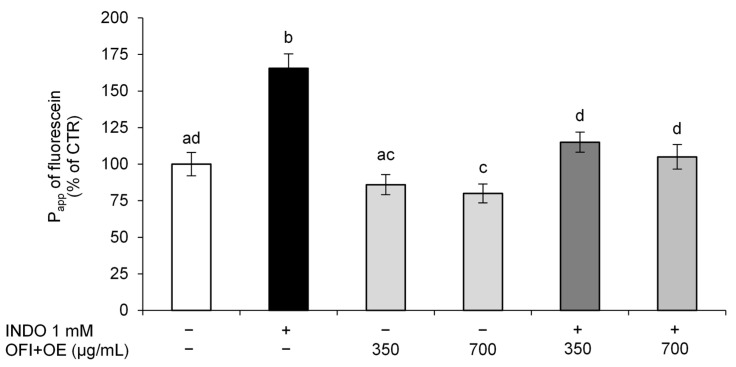
Paracellular permeability of fluorescein. Fully differentiated Caco-2 cells were pre-treated with OFI+OE (350 and 700 μg/mL) for 24 h and then exposed to INDO 1 mM for 24 h. At the end of the treatments, paracellular permeability to fluorescein was evaluated. Cells treated with the vehicle alone (DMSO 0.1%) were used as controls. Results are reported as the percentage of P_app_ against control (%) and expressed as mean ± SD of three independent experiments. Means with the same letter are not significantly different from each other (*p* > 0.05).

After the appropriate treatments, fluorescein paracellular permeability in Caco-2 cell monolayers was evaluated. The results obtained showed that INDO damaged the monolayer integrity, as shown by the increase in the paracellular permeability of fluorescein (Figure 2). On the other hand, pre-treatment with OFI+OE, at both the tested concentrations (350 and 700 μg/mL), dose-dependently improved the integrity of Caco-2 cells monolayers, decreasing the permeability of fluorescein.

**Figure 3:**
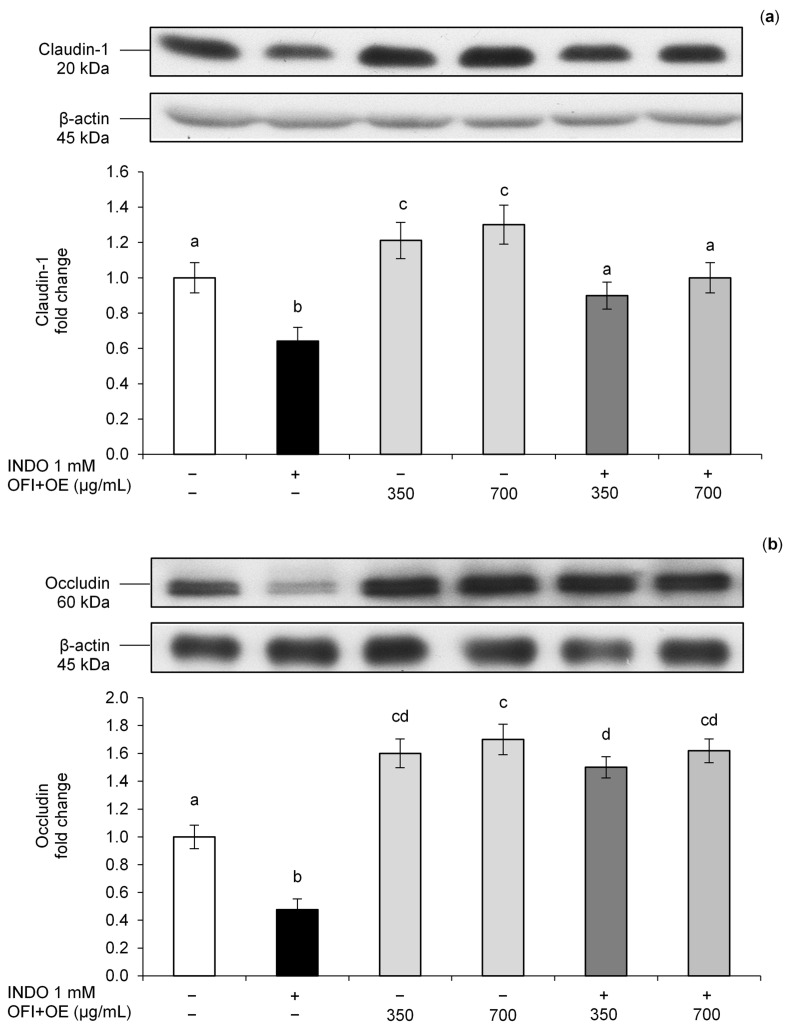
(**a**) Claudin-1 protein levels; (**b**) Occludin protein levels. Caco-2 cells were pre-treated with OFI+OE (350 and 700 μg/mL) for 24 h and then exposed to INDO 1 mM for 24 h. Cultures treated with the vehicle alone (DMSO 0.1%) were used as controls. Claudin-1 and Occludin intensity values were normalized to the corresponding β-actin values. Results are reported as fold change against control and expressed as mean ± SD of three independent experiments. Means with the same letter are not significantly different from each other (*p* > 0.05).

In order to clarify the implication of TJ protein integrity in the alteration of permeability induced by INDO exposure, Claudin-1 and Occludin protein levels were assessed. Cells treated with INDO showed reduced Claudin-1 and Occludin protein levels compared to control cells, demonstrating its injury on the TJ complex (Figure 3a,b). Pre-treatment with OFI+OE enhanced Claudin-1 and Occludin expression in a dose-dependent manner. Interestingly, treatment with only OFI+OE showed a significant increase in Claudin-1 and Occludin levels compared to control cells.

**Figure 4:**
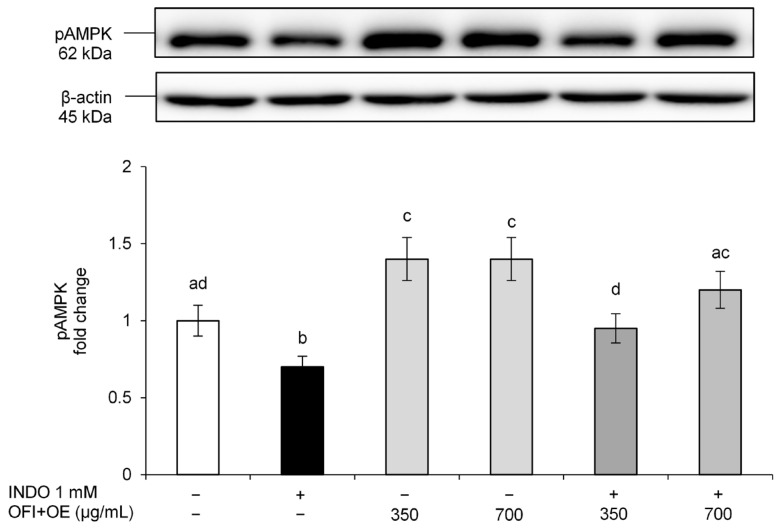
pAMPK protein levels. Caco-2 cells were pre-treated with OFI+OE (350 and 700 μg/mL) for 24 h, and subsequently exposed to INDO 1 mM for 24 h. Cultures treated with the vehicle alone (DMSO 0.1%) were used as controls. pAMPK intensity values were normalized to the corresponding β-actin values. Results are reported as fold change against control and expressed as mean ± SD of three independent experiments. Means with the same letter are not significantly different from each other (*p* > 0.05).

Finally, we examined the activated/phosphorylated AMPK (pAMPK), a protein which plays a pivotal role in apical junction assembly and stability and promotes epithelial barrier functions [58]. INDO reduced pAMPK levels, whereas OFI+OE pre-treatment showed a significant increase in AMPK phosphorylation (Figure 4). Its beneficial effects on intestinal epithelial barrier integrity were demonstrated also by a significant, and dose-dependent, AMPK activation in cells treated only with OFI+OE.

### 3.2. Protective Effect of OFI+OE on the Intracellular Redox Status Alteration Induced by INDO

Oxidative stress is related to the induction and propagation of several gastrointestinal discomforts [59]. A recent study demonstrated that INDO induces cytotoxicity in human intestinal epithelial Caco-2 cells possibly via inhibition of mitochondrial complex I activity, resulting in enhanced production of ROS, endoplasmic reticulum (ER) stress and mitochondria dysfunction [18]. Moreover, oxidative stress and mitochondrial dysfunction have been demonstrated to affect epithelial barrier function, disassembling TJ proteins and enhancing paracellular permeability in vitro [60].

**Figure 5:**
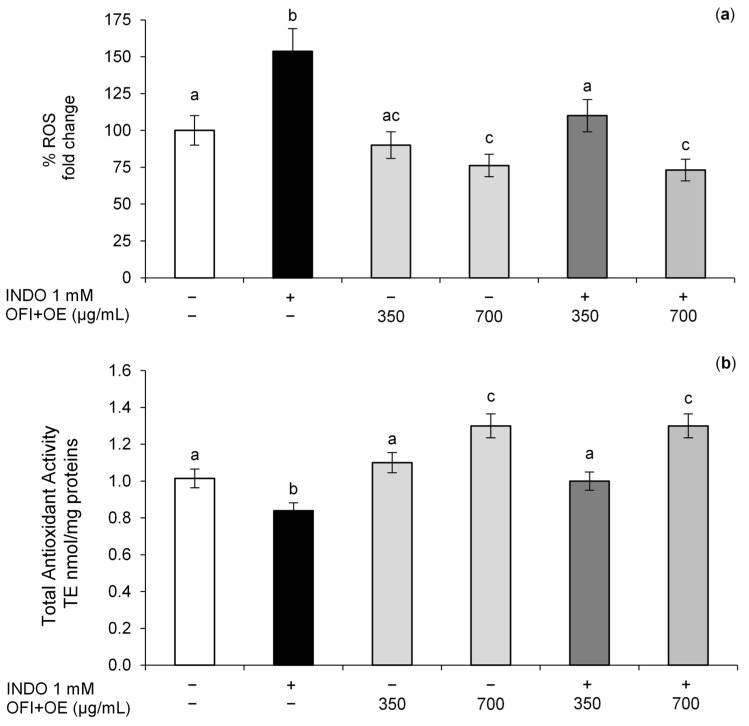
Intracellular redox status. (**a**) ROS levels; (**b**) TAA levels. The Caco-2 monolayers were pre-treated for 24 h with OFI+OE (350 and 700 μg/mL) and exposed to INDO 1 mM for 24 h. Cultures treated with the vehicle alone (DMSO 0.1%) were used as controls. ROS (**a**) and TAA (**b**) levels are reported as % of DCFH-DA fluorescence intensity relative to control and nmoles of Trolox Equivalents/mg of proteins (TE nmol/mg proteins), respectively. Results are expressed as mean ± SD of three independent experiments. Means with the same letter are not significantly different from each other (*p* > 0.05).

In this study, we evaluated OFI+OE effects on INDO-induced oxidative stress in Caco-2 cells by examining ROS and TAA levels, markers of intracellular redox balance. INDO exposure induced enhancement of ROS levels (Figure 5a) and a reduction in TAA values (Figure 5b) compared to control cells, suggesting that INDO was able to induce oxidative stress and imbalance of redox status in the intestinal epithelium. On the contrary, OFI+OE pre-treatment at both tested concentrations (350 and 700 μg/mL) improved the redox balance, as demonstrated by the reduction in the intracellular production of ROS and the increase in TAA values. Moreover, OFI+OE at the highest concentration tested (700 μg/mL) significantly improved the intracellular redox balance in Caco-2 cells not exposed to INDO.

### 3.3. OFI+OE Effects on Apoptosis Pathway

High intracellular ROS levels promote ER stress and mitochondrial dysfunction, leading to apoptotic cell death in Caco-2 intestinal cells [61,62].

In mitochondrial apoptosis, the permeability of the outer membrane is regulated by the balance between anti-apoptotic family proteins (Bcl-2, Bcl-xL, and Mcl-1) and their pro-apoptotic counterparts (BAX, BAK and BAD) [63]. When mitochondrial outer membrane permeability is increased, the Bcl-2/BAX expression ratio decreases, and cytochrome *c* is released into the cytosol and binds to Apaf-1 (apoptotic protease-activating factor 1), leading to activation of the caspase cascade. Then, Caspase-3 is activated and stimulates other proteins and enzymes to break down the cell and form apoptotic bodies, which are finally endocytosed by macrophages and neighbouring cells [64,65].

**Figure 6:**
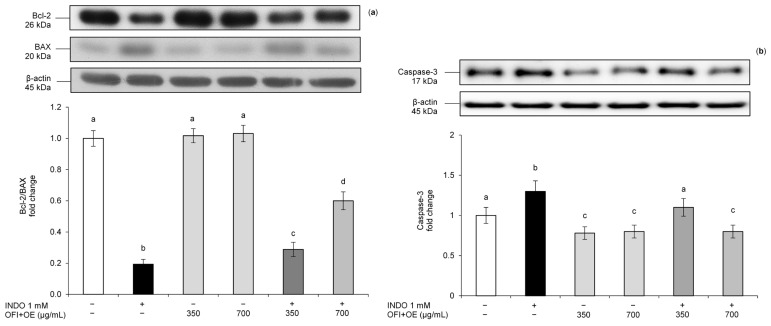
(**a**) Bcl-2/BAX ratio protein expression and (**b**) Caspase-3 protein levels. Caco-2 cells were pre-treated with OFI+OE (350 and 700 μg/mL) for 24 h, and subsequently exposed to INDO 1 mM for 24 h. Cultures treated with the vehicle alone (DMSO 0.1%) were used as controls. (**a**) Bcl-2, BAX, and (**b**) Caspase-3 intensity values were normalized to the corresponding β-actin values. Results are reported as fold change against control and expressed as mean ± SD of three independent experiments. Means with the same letter are not significantly different from each other (*p* > 0.05).

Therefore, we studied the effects of OFI+OE on the INDO-induced apoptosis pathway in Caco-2 cells. Cells incubated with INDO showed an upregulation of pro-apoptotic BAX protein and a reduction in anti-apoptotic Bcl-2 protein, resulting in a lower Bcl-2/BAX expression ratio compared to control cells, confirming that INDO induced cytotoxicity on the intestinal barrier (Figure 6a). On the other hand, pre-treatment with OFI+OE improved the Bcl-2/BAX expression ratio compared to the control cell level. OFI+OE treatment alone did not show any significant effect on Bcl-2 or BAX proteins basal levels.

These results were confirmed by analysis of Caspase-3 levels. This protein, activated at the end of the caspase cascade during the apoptosis process, was activated in Caco-2 cells exposed to INDO, while it was downregulated in cells pre-treated with OFI+OE, reaching protein levels similar to those of control cells (Figure 6b).

### 3.4. Protective Effect of OFI+OE on Indomethacin-Induced NF-κB Pathway Activation

At the intestinal barrier, redox homeostasis and epithelial barrier function are linked to inflammation cascade. The pro-inflammatory effects of INDO have been related to the activation of the nuclear transcription factor kappa B (NF-kB) pathway and the increased expression of proinflammatory cytokines. NF-kB exists in unstimulated cells as a heterodimer (p50 and p65 subunits), bound to IkB protein (inhibitor of kB), which maintains NF-kB in an inactive state. Inflammatory stimuli initiate a signalling cascade, finally leading to the activation of IKK (IkB kinase) which phosphorylates and induces the proteasomal degradation of IkB. Freed NF-kB dimers thus translocate from the cytoplasm to the nucleus, where they bind to specific DNA sequences and regulate the expression of various target genes, including inflammatory response genes [66].

**Figure 7:**
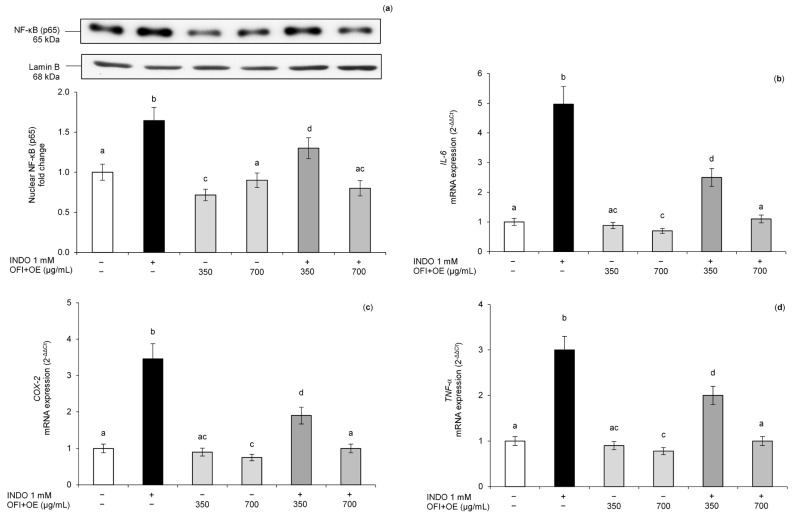
(**a**) Nuclear NF-κB (p65) protein levels; (**b**) *IL-6* gene expression; (**c**) *COX-2* gene expression; (**d**) *TNF-α* gene expression. The Caco-2 monolayers were pre-treated with OFI+OE (350 and 700 μg/mL) for 24 h, and subsequently exposed to INDO 1 mM for 24 h. Cultures treated with the vehicle alone (DMSO 0.1%) were used as controls. Caco-2 nuclear lysates were analysed by Western Blot, and nuclear localization of the p65 protein was evaluated. (**a**) NF-κB (p65) intensity values were normalized to the corresponding Lamin B values. (**b**–**d**) *IL-6*, *COX-2* and *TNF-α* mRNA expression levels were analysed by real-time PCR and data were expressed as 2^−ΔΔCt^ and normalized to control. *18S* rRNA was used as housekeeping gene. Results derived from three independent experiments are reported as mean ± SD. Means with the same letter are not significantly different from each other (*p* > 0.05).

To understand the effect of OFI+OE on the inflammatory response, the activation of the NF-kB pro-inflammatory pathway was evaluated by determination of p65 nuclear localization through Western Blot. Exposure to 1 mM INDO for 24 h activated the NF-kB pathway, leading to high levels of p65 in the nuclear protein lysates of Caco-2 cells (Figure 7a). Pre-treatment with OFI+OE 350 and 700 μg/mL reduced p65 nuclear concentration compared to values measured in control cells, in a dose-dependent manner. Interestingly, cells exposed to only OFI+OE 700 μg/mL showed significantly lower p65 nuclear levels compared to control cells.

After NF-kB pathway activation, p65/p50 dimers translocate to the nucleus and bind to specific DNA sequences, thus modulating gene expression of various pro-inflammatory genes [67], contributing to intestinal inflammation. To confirm this transcriptional activity, we evaluated mRNA expression of *IL-6*, *COX-2*, and *TNF-α*, which are pro-inflammatory genes regulated by the NF-kB pathway, using real-time PCR. Caco-2 cells exposed to INDO showed increased levels of *IL-6*, *COX-2* and *TNF-α* compared to control cells, while pre-treatment with OFI+OE dose-dependently reduced their mRNA values (Figure 7b–d). OFI+OE treatment alone showed only a modest and non-significant effect on the already low basal levels of *IL-6*, *COX-2*, and *TNF-α* gene expression. These results confirmed the protective effect of OFI+OE against intestinal inflammation induced by INDO exposure.

## 4. Discussion

NSAIDs are frequently associated with severe adverse effects affecting GI mucosa, and although PPIs are effective in protecting from NSAID-induced gastropathy, they are useless against NSAID-induced enteropathy [2,13], characterized by alteration of the integrity of epithelial TJs and increased permeability of the intestinal epithelial barrier [9]. The cytotoxic phenomena related to the adverse effects of NSAIDs in the GI tract are associated with mitochondrial dysfunction, oxidative stress, and apoptosis, as previously shown in different in vitro and in vivo studies [6,68]. Therefore, alterations of the epithelial barrier function can represent an important risk factor for developing intestinal disorders, such as Crohn’s disease, ulcerative colitis, enteritis, celiac disease, type 1 diabetes, and infections [5].

The aim of this work was to investigate the in vitro beneficial effects of a commercially available standardized extract (OFI+OE), made through blending *O. ficus-indica* (L.) Mill. cladodes juice extract and *O. europaea* L. leaves aqueous extract, against NSAID-induced alterations on the intestinal epithelial barrier.

*O. ficus-indica* (L.) Mill. cladodes are well-recognized for their mucoadhesive properties and mucoprotective action, thanks to the presence of several polysaccharides, which are useful in the treatment of mucosal injuries [23]. The capability of *Opuntia* cladodes polysaccharides to adhere to cell surfaces and create a physical barrier against chemical stressors was also demonstrated in some in vitro intestinal epithelial models [37]. Other recent studies reported the protective activity of different plant and mushroom polysaccharides on intestinal TJs, thus improving gut epithelial barrier function and preventing intestinal barrier injury [69,70,71,72,73,74,75]. One has to mention that interactions between polyphenols and polysaccharides have been utilized for targeted release of bioactive compounds [76,77,78,79]. Moreover, *Opuntia* cladodes contain several bioactive polyphenols, in particular piscidic acid, and *O. europaea* L. leaf extracts are widely studied for radical scavenger, antioxidant, and anti-inflammatory activities, thanks to the presence of polyphenols [37,46,80]. The main polyphenolic components of the extract tested in our study were oleuropein and luteolin glycosides, together with lower amounts of apigenin glycoside, verbascoside, and hydroxytyrosol (Figure S1 and Table S1) [81]. The protective effects of *O. europaea* leaf extract were previously reported in mice and rat models of colitis [82,83], whereas Elmaksoud et al. [46] demonstrated that hydroxytyrosol alleviates intestinal inflammation in experimental ulcerative colitis, and Min et al. [84] reported that luteolin-7-O-beta-D-glucuronopyranoside considerably decreases the size of gastric lesions. Furthermore, Larussa et al. [85] reported the anti-inflammatory properties of oleuropein in colonic samples from patients with ulcerative colitis.

Given their high annual productivity, accompanied by the high amount of by-products generated and the derived environmental concerns, both plants can represent an economic and suitable source for the isolation of bioactive compounds, leading to considerable pharmacological interest. In fact, olive leaves constitute the most abundant waste product of the olive industry (8–10% of the total weight of olives subjected to milling) and agronomic practices (with around 25 kg of branches and leaves derived annually from pruning each olive tree) [86]. Regarding *O. ficus-indica*, the fruit is the main product. However, the mature plant has a median yield of 250 cladodes that, although used as fodder and in some countries as human food, are in part disposed of in landfills [87,88]. Therefore, both olive leaves and *Opuntia* cladodes could be interesting from the perspective of the virtuous use of agricultural waste.

Herein, we used Caco-2 cells as a model of the intestinal epithelial barrier, and INDO, an NSAID generally used in therapy for its anti-inflammatory properties, as a drug model to simulate in vitro a condition of intestinal injury. In this model, both the extract as well as INDO were added in the apical compartment of transwell inserts with fully differentiated Caco-2 cells to mimic oral administration and subsequent absorption from the intestinal lumen to blood circulation.

Initially, we focused on the protective activities of OFI+OE on INDO-induced intestinal epithelial barrier function alteration. The intestinal epithelium constitutes a physical barrier that regulates the selective paracellular permeability to molecules and maintains homeostasis. Mucosal barrier function is preserved by different elements, such as the microbiota, mucus layer, mucosal immune system, and epithelium [89]. Epithelial cells are connected by an apical intercellular complex, constituted by adherens junctions, desmosomes and TJs, which are responsible for the passage of solutes through pores. INDO has been shown to alter intestinal permeability by disassembling TJ proteins. Through TEER measurement and fluorescein paracellular permeability evaluation, we observed that INDO was able to cause epithelial barrier injury, as shown by a decrease in TEER values and an increase in permeability to fluorescein, whereas pre-treatment with OFI+OE protected intestinal cells from INDO-induced damage, leading to higher TEER values and lower permeability. Moreover, the extract also improved basal intestinal epithelial barrier function in cells not exposed to INDO (Figure 1 and Figure 2).

Claudin-1 and Occludin are the main apical junction proteins regulating the passage of molecules from the luminal compartment to the systemic circulation, and the activation/phosphorylation of AMPK is also required for the assembly and stability of TJs [58]. In our experiments, we clarify the implication of TJs integrity alteration in the negative effects of INDO on epithelial barrier function, given that exposure to this NSAID reduced Claudin-1, Occludin, and pAMPK levels. OFI+OE beneficial effects on monolayer integrity were demonstrated by consistent and dose-dependent increases in TJ protein levels and activation of the AMPK pathway (Figure 3 and Figure 4).

Several studies suggested that INDO exposure can induce oxidative stress in intestinal epithelial cells, leading to ER stress, mitochondria dysfunction and cell death [18]. The mechanism underlying INDO cytotoxicity is not well known but NSAIDs have been demonstrated to uncouple oxidative phosphorylation, decrease mitochondrial membrane potential, and reduce both mitochondrial oxygen consumption and ATP synthesis [90]. Moreover, oxidative stress and correlated production of ROS, such as hydrogen peroxide and nitric oxide, have been demonstrated to disrupt TJ proteins and alter paracellular permeability in vitro. Therefore, oxidative stress has a key role in INDO-induced cytotoxicity in the GI tract [91]. Phytochemicals with antioxidant and anti-inflammatory activities are promising in preventing the activation of oxidative and inflammatory pathways during pathological states [92]. *O. ficus-indica* (L.) Mill. cladodes extract is generally noted for its mucoprotective properties, and in combination with *O. europaea* L. leaves, rich in polyphenols, may represent a potential therapy against NSAID-induced oxidative stress. Moreover, the interaction between polyphenols and polysaccharides may play a crucial role in the digestive tract, favouring their delivery to the GI mucosa [93,94,95]. A previous study showed that olive leaf extract has antioxidant activity greater than vitamins C and E, thanks to the combined effects of its flavonoids, oleuropeosides, and substituted phenols [80], which scavenge ROS and inhibit lipid peroxidation. Oleuropein also possesses the ability to inhibit the production of ROS and RNS (reactive nitrogen species) in vitro cell-free models and in cultured cells [96,97]. In this study, we evaluated the effects of OFI+OE on intracellular redox balance by measurement of intracellular ROS concentrations and TAA. OFI+OE pre-treatment improved the redox balance in intestinal cells exposed or not to INDO, reducing intracellular production of ROS, and improving antioxidant activity (Figure 5a,b), suggesting the scavenging activity of the extract.

High intracellular ROS are crucial in triggering apoptotic cell death, through the mitochondrial apoptosis pathway mediated by Bcl-2/BAX factors [98,99]. It consists of the opening of the mitochondrial outer membrane pore regulated by Bcl-2 family proteins [100]. Anti-apoptotic Bcl-2 family proteins, such as Mcl-1, Bcl-2, and Bcl-xL, prevent pore formation, while pro-apoptotic BH3-only proteins activate BAX and BAK, leading to the formation of mitochondrial pore and release of cytochrome *c* [101]. In turn, these events activate Caspase-9 and executioner caspases, such as Caspase-3 and Caspase-7, until the formation of apoptotic bodies. According to this, we evaluated the Bcl-2/BAX ratio and Caspase-3 protein levels. The results confirmed INDO cytotoxicity on the intestinal barrier, shown by higher Caspase-3 and BAX levels and downregulation of Bcl-2 protein, and thus to a lower Bcl-2/BAX expression ratio. OFI+OE showed protective effects on INDO-induced apoptosis, decreasing pro-apoptotic markers BAX and Caspase-3 and increasing anti-apoptotic factor Bcl-2 (Figure 6a,b). Elmaksoud et al. [46] previously demonstrated, besides the anti-inflammatory and antioxidant effects, with downregulation of pro-inflammatory cytokines, a reduction in colon malondialdehyde, myeloperoxidase and nitric oxide levels, and an increase in superoxide dismutase, catalase, and glutathione peroxidase levels, also the anti-apoptotic activity of an olive leaf extract standardized with 25% hydroxytyrosol in an experimental model of ulcerative colitis, reporting BAX downregulation and Bcl-2 upregulation.

Increasing interest in plant polyphenols results from their antioxidant power, which is involved in the prevention of the inflammatory process [92]. In particular, oleuropein has demonstrated high antioxidant activity with anti-inflammatory properties [45]. This is probably thanks to its ability to chelate metal ions, such as Cu and Fe, responsible for free radical generation [102,103], and its ability to inhibit several inflammatory enzymes. It has shown intestinal anti-inflammatory effects in various experimental models of colitis, thanks to its antioxidant power and modulation of immune response, recovering from intestinal epithelial cell injury [104,105]. Hydroxytyrosol is also endowed with strong antioxidant activity related to the electron-donating capacity of its hydroxyl groups, its ability to form stable hydrogen bonds with phenoxyl radicals, and its metal-chelator activity [106,107]. Additionally, luteolin and luteolin glycosides have shown important antioxidant activities, acting directly as free radical scavengers, but also by inducing endogenous antioxidant cell defences through activation of the Nrf2 pathway [108,109,110].

To clarify the effects of OFI+OE on the inflammatory response, we evaluated the activation of the NF-kB pro-inflammatory pathway by determining p65 nuclear localization. The data proved the anti-inflammatory effect of OFI+OE extract, inhibiting the NF-kB pathway induced by INDO exposure (Figure 7a). To confirm this evidence, we focused on the modulation of pro-inflammatory gene expression, such as *IL-6*, *COX-2*, and *TNF-α*, which are upregulated when the p65 subunit translocates from the cytoplasm to the nucleus and binds specific DNA sequences. Pre-treatment with OFI+OE reduced *IL-6*, *COX-2*, and *TNF-α* gene expression compared to cells treated with INDO (Figure 7b,c). The improvement of the cytokine profile can reduce the activation and migration of leukocytes to the inflamed region and their dangerous effects on intestinal tissue [111,112]. Moreover, OFI-OE reduced p65, *IL-6*, *COX-2*, and *TNF-α* gene expression levels also compared to control cells not exposed to INDO (Figure 7). Oxidative stress can induce the activation of ROS-dependent cell signalling pathways, such as the NF-kB pathway [21]. Thus, previously shown oxidative stress induced by INDO (Figure 5) may explain the activation of the NF-kB pathway induced by NSAID exposure. At the same time, the anti-inflammatory activity of OFI+OE could be attributed to its ability to improve intracellular redox status, reducing ROS levels and improving TAA.

## 5. Conclusions

In this study, we demonstrate the in vitro protective effects of a combined extract of *O. ficus-indica* (L.) Mill. cladodes and *O. europaea* L. leaves against NSAID-induced intestinal epithelial barrier injury. The beneficial effects of this extract are correlated to the ability of its constituents to maintain and improve intestinal membrane integrity, protecting cells against oxidative stress induced by INDO exposure. Moreover, the results clarify the molecular mechanisms involved in the modulation of oxidative, inflammatory, and apoptotic pathways. In conclusion, although we recognize that these in vitro findings may not accurately represent the impact of OFI+OE extract on human tissues and that further studies on in vivo experimental models are required to validate these data, the present study provides a basis for the potential use of this natural combined extract as a strategy for treating and preventing damage at the intestinal mucosa level.

## Data Availability

The original contributions presented in this study are included in the article/Supplementary Material. Further inquiries can be directed to the corresponding author (F.C.).

## Supplementary Materials

The following supporting information can be downloaded at: https://www.mdpi.com/article/10.3390/antiox13121507/s1, Figure S1: HPLC-DAD polyphenolic profile the OFI-OE extract; Table S1: Polyphenolic compounds identified in the OFI-OE extract; Figure S2: Cell viability evaluation [113].

## Abbreviations

The following abbreviations are used in this manuscript:

ABTS	2,2-azinobis-(3-ethybenzothiazoline-6-sulfonic acid)
AMPK	5′ adenosine monophosphate-activated protein kinase
DCFH-DA	dichloro-dihydro-fluorescein diacetate
DMEM	Dulbecco’s modified eagle’s medium
DMSO	dimethyl sulfoxide
DPBS	Dulbecco’s phosphate-buffered saline
ER	endoplasmic reticulum
GI	gastrointestinal
INDO	indomethacin
NSAIDs	nonsteroidal anti-inflammatory drugs
OFI+OE	*Opuntia ficus-indica* (L.) Mill. cladodes and *Olea europaea* L. leaf extract
PPIs	proton pump inhibitors
ROS	reactive oxygen species
TAA	Total Antioxidant Activity
TEER	Trans-Epithelial Electrical Resistance
TJs	tight junctions
