# Supplementary material for: In Vitro Protective Effects of a Standardized Extract of Opuntia ficus-indica (L.) Mill. Cladodes and Olea europaea L. Leaves Against Indomethacin-Induced Intestinal Epithelial Cell Injury
Source: Antioxidants (Basel). 2024 Dec 10;13(12):1507. doi: 10.3390/antiox13121507 (PMC11673993; doi:10.3390/antiox13121507)
Supplement: Supplementary file 1 [file antioxidants-13-01507-s001.zip › antioxidants-3310188-supplementary.pdf]

Supplementary material

Extract specification

The extract (Mucosave™ FG, Batch Number: 05202210-06, provided by Bionap Srl, Catania, Italy) is obtained by extraction from *Olea europaea* L. [Oleaceae] leaves in water at 50 °C, followed by purification with resin, and by filtration of cold press juice from *Opuntia ficus-indica* L. [Cactaceae]. The two extracts are mixed (32-35% w/w of *Opuntia ficus-indica* cladodes extract, and 23-25% w/w of *Olea europaea* L. leaves extract) and dried by spray drier, adding maltodextrins (40-45%). This commercial extract is standardized to contain 3.7–4.3% (w/w) total polyphenols (as luteolin 7-O-glucoside), and 18–28% (w/w) total polysaccharides (as glucose), with maltodextrins as an excipient. The batch used contains 3.9% of total polyphenols, 27% of total polysaccharides, and 45% (w/w) of maltodextrins.

HPLC-DAD polyphenolic profiling

The powdered samples were dissolved in 1 mL of dimethylformamide /water solution (9:1) with a final concentration of 10 mg/mL. HPLC-DAD analysis was carried out in duplicate using an HPLC 1100 Infinity (Agilent Technologies, Santa Clara, CA, USA), equipped with a diode array detector (DAD) and with an Ascentis Express C 18 column (150 x 4.6 mm i.d., 2.7 μm). The mobile phases consisted of H_2O/H_3PO_4 (99:1, solvent A) and $MeOH/ACN/H_3PO_4$ (49.5:49.5:1, solvent B) with the following gradient elution program: concentration solvent A of 95% going to 77% (0-31 min), maintain 77% (31–34 min), 74% (34-60 min), 60% (60-85 min), 20% (85-90 min), 0% (90-92 min), maintain 0% (92–98 min), and 95% (98–105 min). The column temperature was maintained at 25 °C. The flow rate was 1 mL/min and the injection volume was 5 μL. The profile chromatograms were recorded from 190 to 500 nm. The traces, recorded at 280 nm and 346 nm, are reported in Figure S1 and show the 11 main compounds identified (Table S1). The extract contains 3.9 % w/w luteolin-7-O-glucoside and derivates, as determined using calibration curves with the closest appropriate standard.

The HPLC-grade solvents, methanol, acetonitrile, water, dimethylformamide, and phosphoric acid were obtained from Carlo Erba Reagenti (Milano, Italy). Reference compounds were obtained from PhytoLab GmbH & Co. (Vestenbergsgreuth Germany).

Figure S1. HPLC-DAD polyphenolic profile the OFI-OE extract (MucosaveTM FG, Batch Number: 05202210-06). The numbers indicating peaks refer to the identified compounds.
[1] Hydroxytyrosol, [2] Piscidic acid; [3] Tyrosol; [4] Luteolin 7-O-rutinoside, [5] Luteolin 7-O-glucoside, [6] Verbascoside, [7] Apigenin 7-O-rutinoside, [8] Apigenin 7-O-glucoside, [9] Luteolin 4-O-glucoside, [10] Apigenin 7-O-glucuronide, [11] Oleuropein.

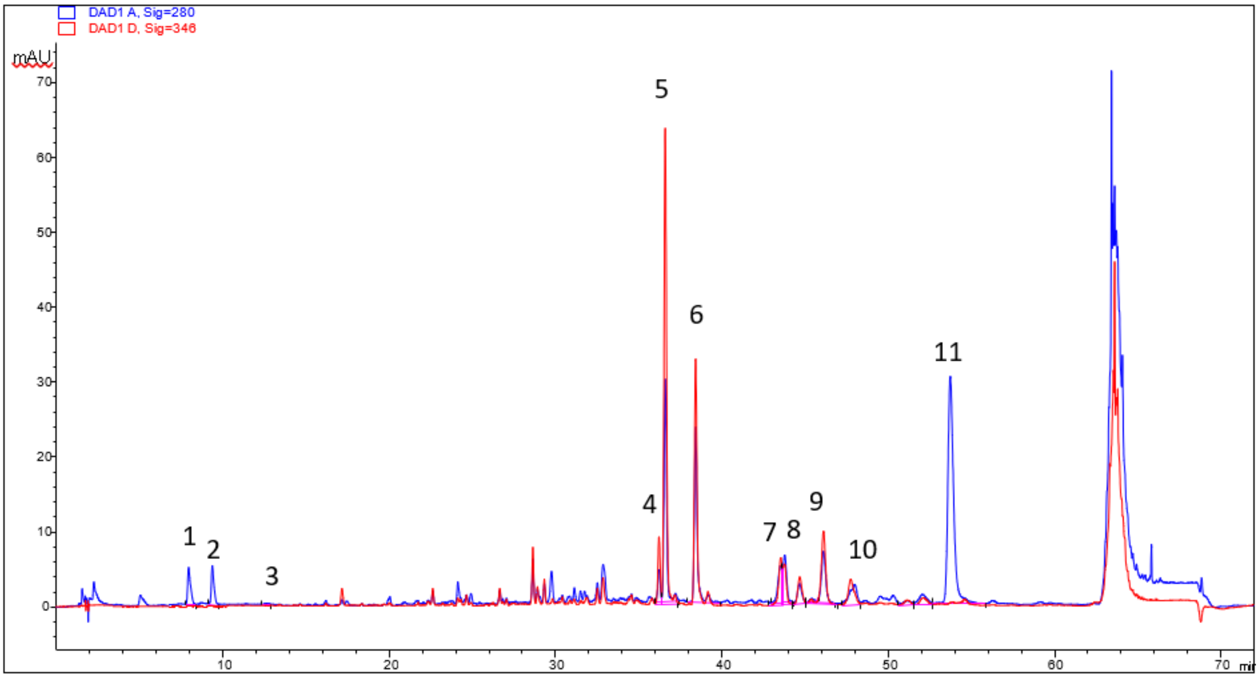

Table S1. Polyphenolic compounds identified in the OFI-OE extract (MucosaveTM FG, Batch Number: 05202210-06).

Peak N.	*Compound*	*Wavelength λ (nm)*	*Retention Time (min)*
1	Hydroxytyrosol	280	7.96
2	Piscidic acid	280	9.37
3	Tyrosol	280	12.69
4	Luteolin 7-O-rutinoside	346	36.21
5	Luteolin 7-O-glucoside	346	36.58
6	Verbascoside	346	38.40
7	Apigenin 7-O-rutinoside	346	43.49
8	Apigenin 7-O-glucoside	346	43.76
9	Luteolin 4-O-glucoside	346	45.37
10	Apigenin 7-O-glucuronide	346	46.10
11	Oleuropein	280	53.72

Cell viability evaluation

The biocompatibility of *Opuntia ficus-indica* (L.) Mill. and *Olea europaea* L. combined extract (OFI+OE) was assessed on human intestinal epithelial cells Caco-2 using the Sulforhodamine B (SRB) assay [1]. Cells were seeded at 4 x 10^4 per cm^2 in 12-well plates (Greiner Bio-One, Italy) and grown in Dulbecco's modified eagle's medium (DMEM), supplemented with 10% FBS, 4 mM L-glutamine, 1% non-essential amino acids, 100 U/mL

penicillin, and 100 μg/mL streptomycin, at 37 °C in humidified atmosphere of 5% CO_2. Cells were cultured for 18 days post-confluence to obtain fully differentiated cells [2, 3]. Then, monolayers were treated for 24 h with different concentrations of OFI+OE extract (200-350-500-700-850-1000 μg/mL) added to the cell culture medium. Control cells were treated with the vehicle alone. At the end of time exposure, the cells were fixed using 10% trichloroacetic acid (w/v) for 1 h at 4 °C and then washed twice with water and incubated with sulforhodamine B (0.4% w/v in 1% acetic acid) for 30 min at RT, followed by four washes with 1% acetic acid. The dye trapped in the cells was dissolved in 10 mM Tris-base solution and the absorbance was measured at 565 nm using a microplate reader (GloMax® Discover System-TM397). The results are reported as the percentage of cell viability against untreated cells used as controls (%).
OFI+OE did not show significant toxicity until the highest tested concentration (1000 μg/mL), thus in this model, the extract was used at not-toxic 350 and 700 μg/mL doses (Figure S2).

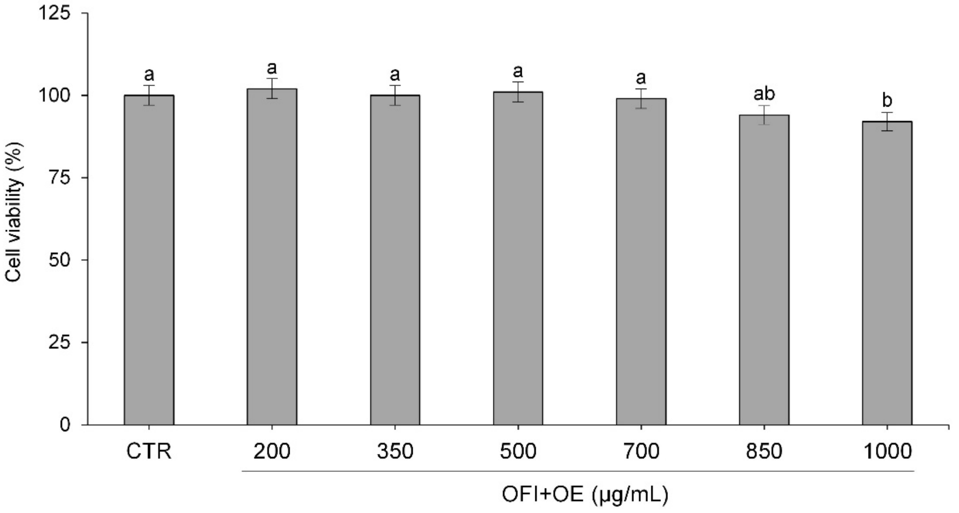

Figure S2. Cell viability evaluation. Caco-2 cells were exposed to OFI+OE (200, 350, 500, 700, 850, and 1000 μg/mL) for 24 h. Cultures treated with the vehicle alone were used as controls. The results are reported as the percentage of cell viability against control (%) and expressed as mean ± SD of three independent experiments. Means with the same letter are not significantly different from each other ($p > 0.05$).

References

1. Vichai, V.; Kirtikara, K., Sulforhodamine B colorimetric assay for cytotoxicity screening. Nat. Protoc. 2006, 1, 3, 1112-6.
2. Ferrari, D.; Speciale, A.; Cristani, M.; Fratantonio, D.; Molonia, M. S.; Ranaldi, G.; Saija, A.; Cimino, F., Cyanidin-3-O-glucoside inhibits NF-kB signalling in intestinal epithelial cells exposed to TNF-alpha and exerts protective effects via Nrf2 pathway activation. Toxicol. Lett. 2016, 264, 51-58.
3. Ferrari, D.; Cimino, F.; Fratantonio, D.; Molonia, M. S.; Bashllari, R.; Busa, R.; Saija, A.; Speciale, A., Cyanidin-3-O-Glucoside Modulates the In Vitro Inflammatory Crosstalk between Intestinal Epithelial and Endothelial Cells. Mediators Inflamm. 2017, 2017, 3454023.
